# Supplementary material for: Structural basis of sodium ion-dependent carnitine transport by OCTN2
Source: Nat Commun. 2025 Nov 29;17:181. doi: 10.1038/s41467-025-66867-6 (PMC12780140; doi:10.1038/s41467-025-66867-6)
Supplement: Supplementary file 1 — Supplementary Information [file 41467_2025_66867_MOESM1_ESM.pdf]

SUPPLEMENTARY INFORMATION

Structural basis of sodium ion-dependent carnitine transport by OCTN2

James S. Davies[1,#], Yi C. Zeng[1,2,#,*], Chelsea Briot[3], Simon H.J. Brown[4,5], Renae M. Ryan[3,6,*] & Alastair G. Stewart[1,2,*]

Supplementary Table 1. Cryo-EM data collection, refinement and validation statistics

	**OCTN2 inward-facing**<br>EMDB-**71735**<br>PDB **9PMD**	**OCTN2 occluded**<br>EMDB- **71540**<br>PDB **9PDQ**	**OCTN2 ipratropium**<br>EMDB-**71597**<br>PDB **9PFB**
**Data collection and processing**			
Magnification	105,000 x	105,000 x	105,000 x
Voltage (kV)	300	300	300
Electron exposure (e–/Å^2)	81	82	79
Defocus range (μm)	-1, -1.5	-1, -1.5	-1, -1.5
Pixel size (Å)	0.83	0.83	0.83
Symmetry imposed	*C*1	*C*1	*C*1
Initial particle images (no.)	8,483,360	6,417,208	10,039,029
Final particle images (no.)	94,746	181,269	382,921
Map resolution (Å)<br>FSC threshold	2.99<br>0.143	2.72<br>0.143	3.06<br>0.143
**Refinement**			
Initial model used (PDB code)	AlphaFold model (AF-O76082-F1-v4)		
**Model composition**			
Non-hydrogen atoms	4193	4267	4215
Protein residues	528	528	528
Ligands	1	5	2
***B* factors (Å^2)**			
Protein	108.13	100.94	97.15
Ligands	99.44	156.82	73.32
**R.m.s. deviations**			
Bond lengths (Å)	0.002	0.003	0.003
Bond angles (°)	0.566	0.559	0.637
**Validation**			
MolProbity score	1.2	1.2	1.1
Clashscore	4.27	4.32	3.06
Poor rotamers (%)	0.87	0.22	0.65
**Ramachandran plot**			
Favoured (%)	98.67	98.11	99.05
Allowed (%)	1.33	1.89	0.95
Disallowed (%)	0	0	0

Supplementary Figure 1. hOCTN2 purification and cryo-EM data processing & analysis workflow. (**a**) Size-exclusion trace and SDS-PAGE gel (**b**) Representative micrograph and corresponding power spectrum (OCTN2-occluded). (**c**) OCTN2 inward-facing processing workflow including local resolution estimate (**d**) Gold standard & conical Fourier shell correlation curves.

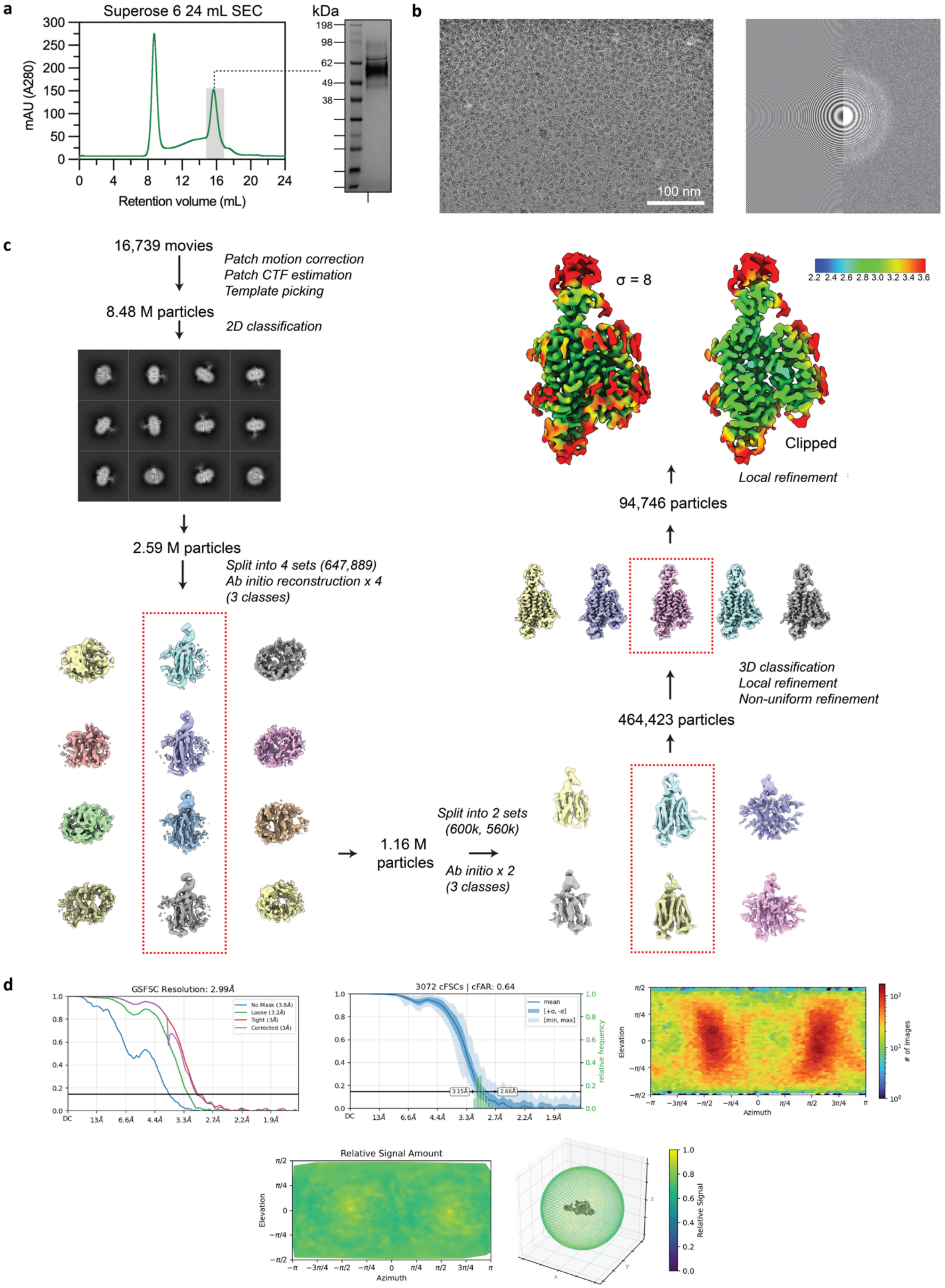

Supplementary Figure 1 [continued] (**a**) OCTN2 occluded processing workflow including local resolution estimate (**b**) Gold standard & conical Fourier shell correlation curves.

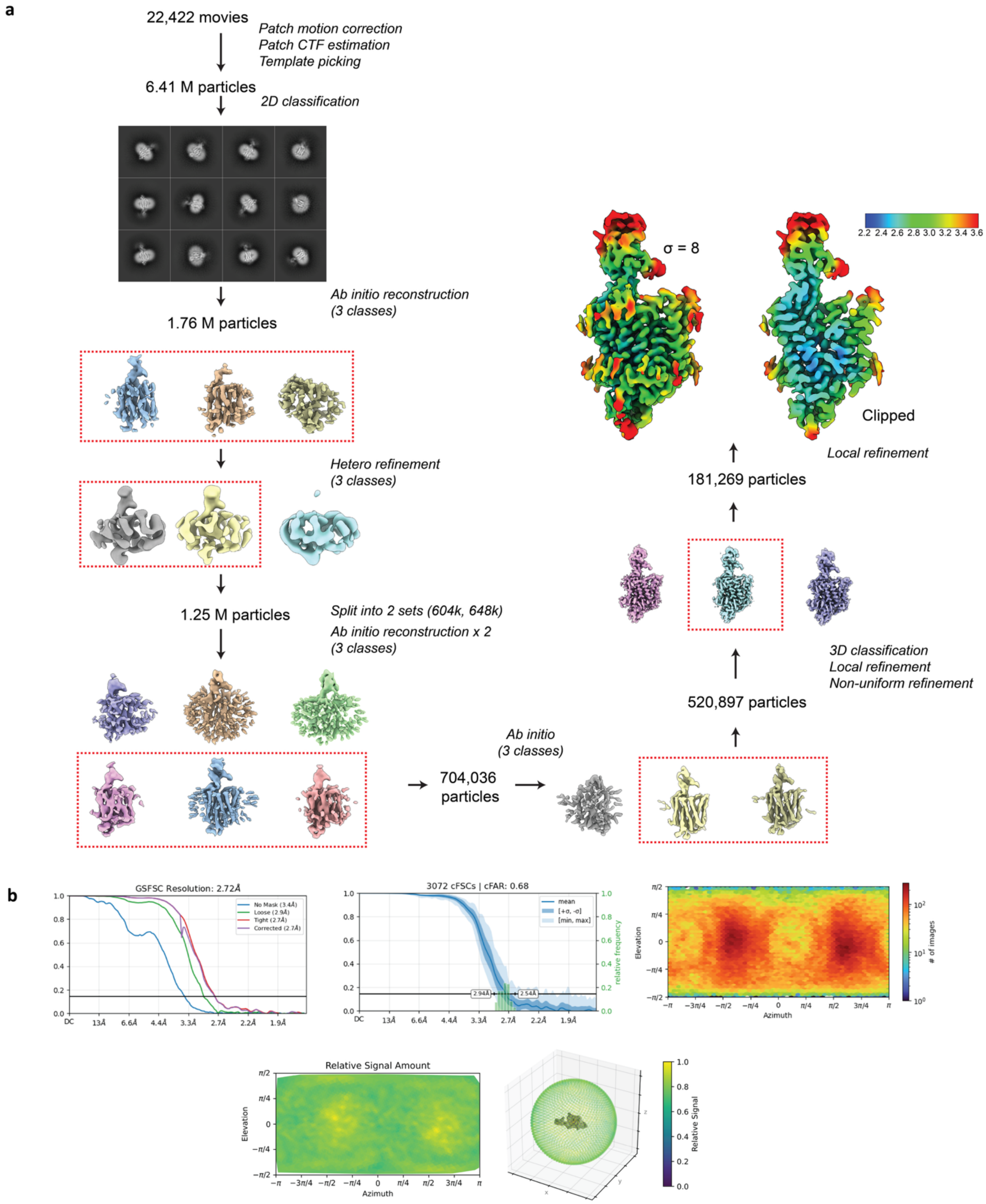

Supplementary Figure 1 [continued] (**a**) OCTN2-ipratropium processing workflow including local resolution estimate (**b**) Gold standard & conical Fourier shell correlation curves

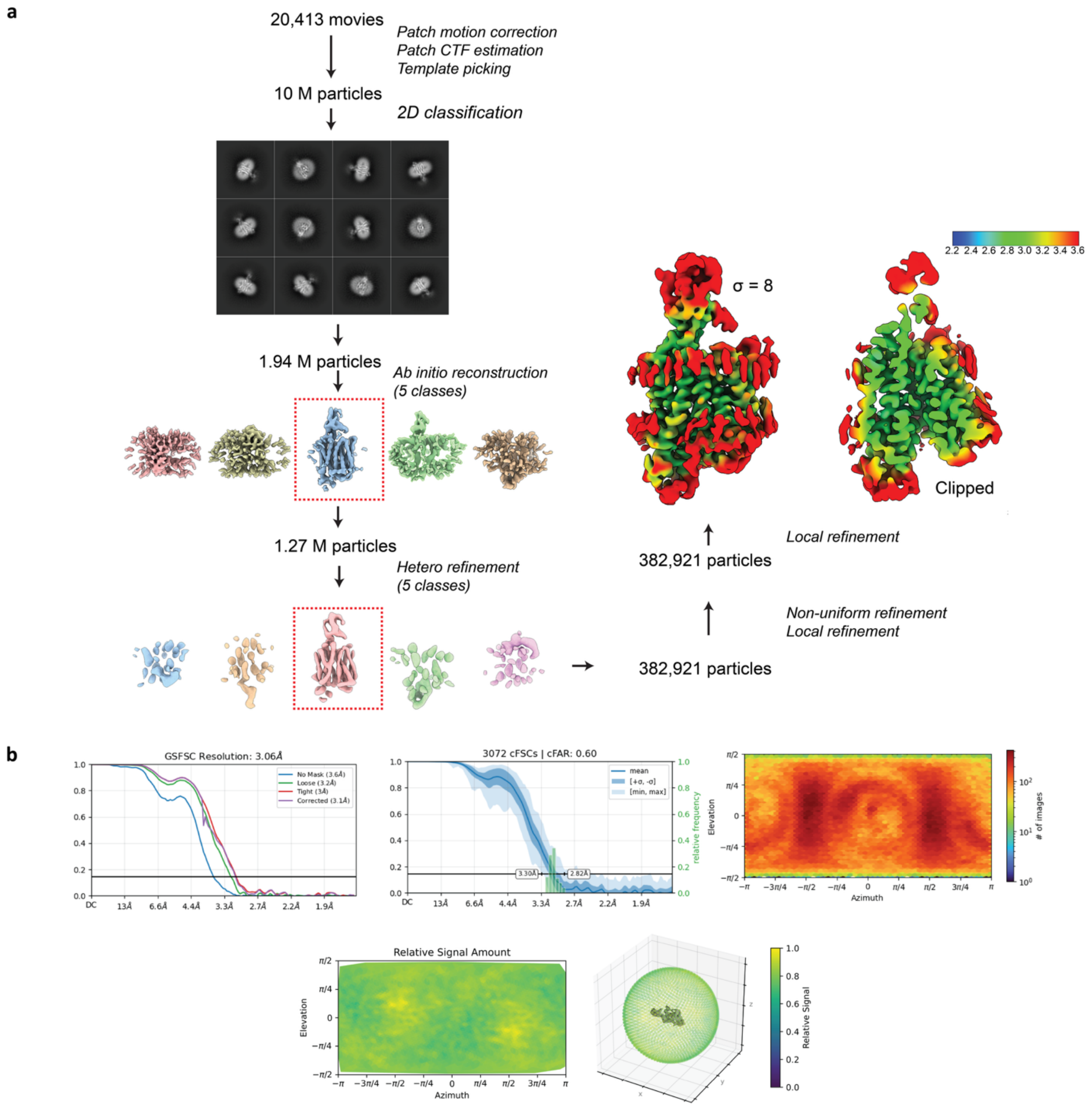

Supplementary Figure 2. Cryo-EM densities of OCTN2 helices, peripheral domains and ligands.

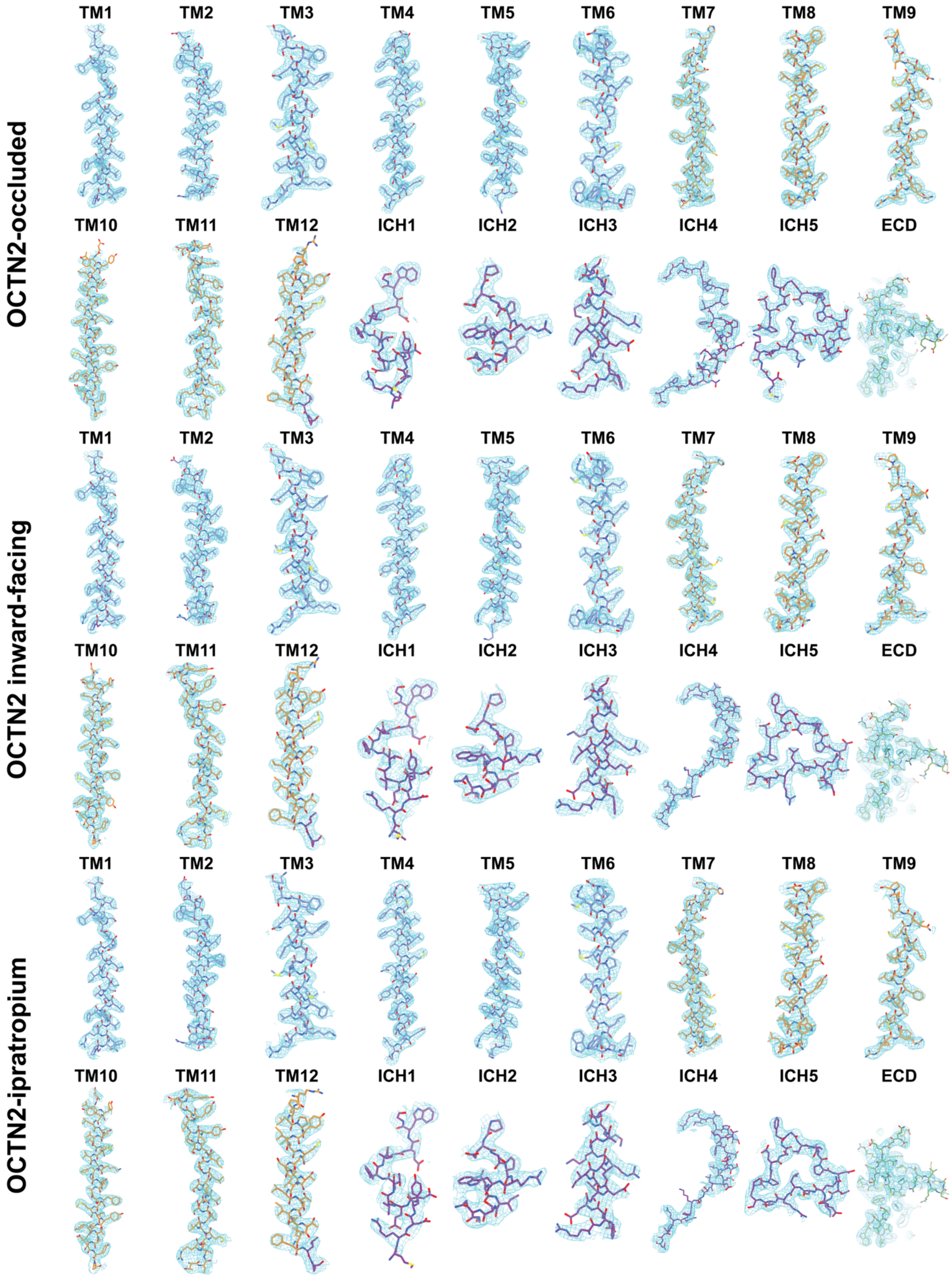

Supplementary Figure 3. Cryo-EM density corresponding to modelled water molecules and Na^+ within the Na^+-binding cavity, and ligands within the substrate-binding cavity (surface representation). Sharpened maps were automatically generated by *cryoSPARC* and are available as supplementary maps in each EMDB deposition. Density is contoured at map levels calculated by *ChimeraX* as indicated.

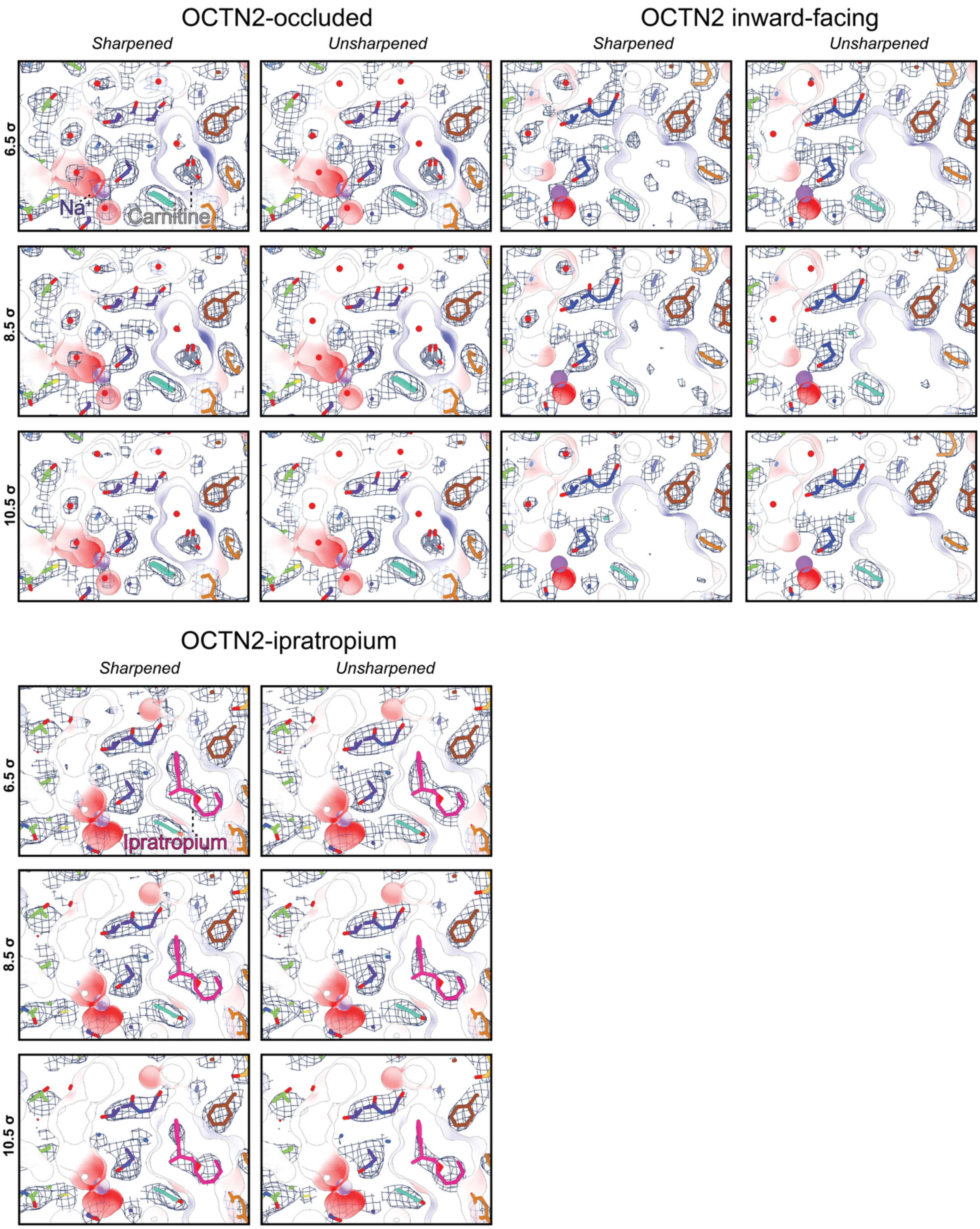

Supplementary Figure 4. Cryo-EM density corresponding to N-linked glycans decorating the extracellular domain. The outline of the OCTN2 occluded map at 4σ (as calculated by *ChimeraX*) is shown in white, while the map at 7σ is shown in green. The modelled GlcNAc residue at each position is shown in pink.

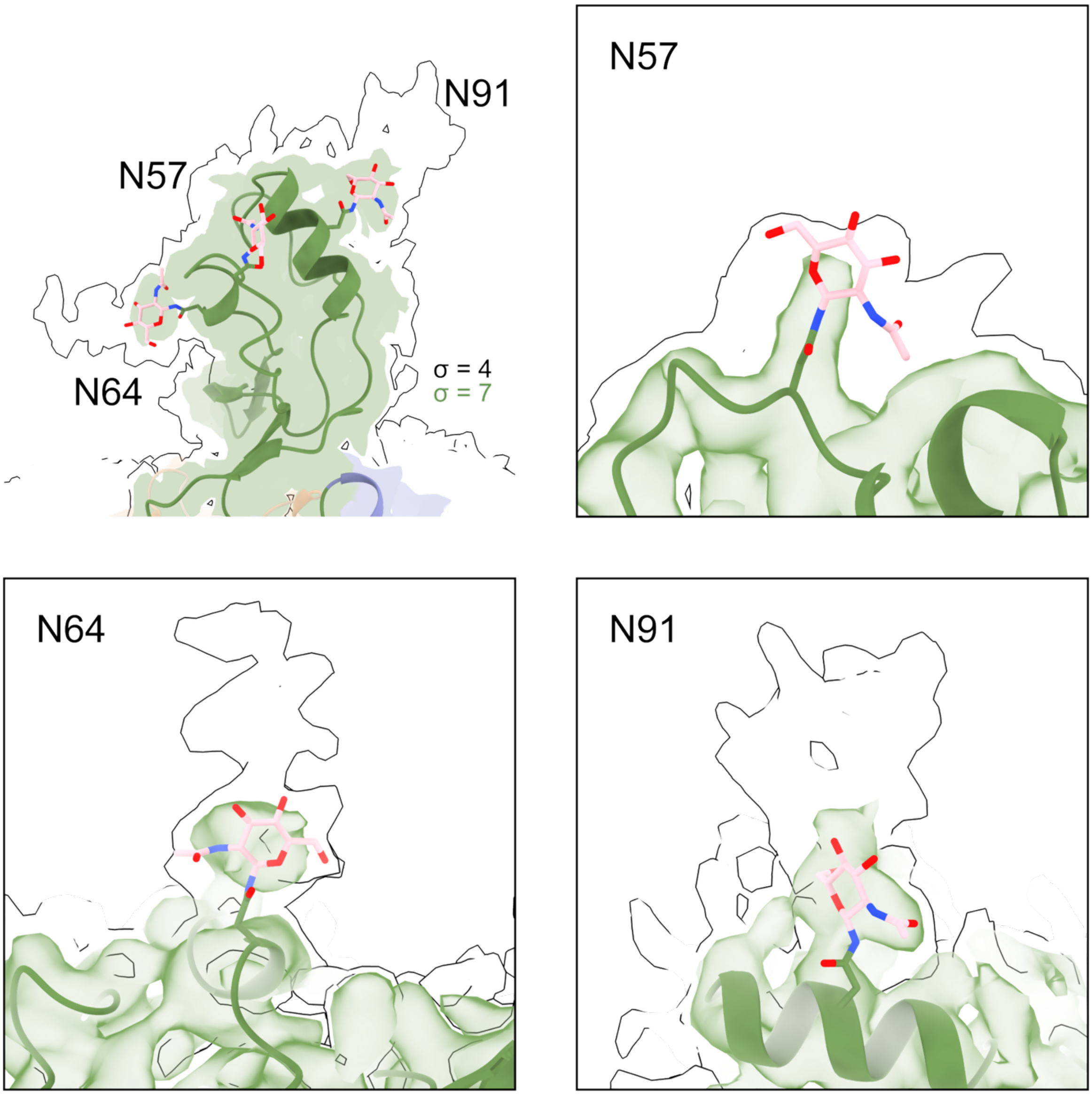

Supplementary Figure 5. (**a**) Cryo-EM density for interactions near the carnitine-binding site. + and **-** symbols indicate the charged sides of carnitine within the cavity. Densities are contoured at 7.5σ, and in the case of the water network, the sharpened map was used. (**b**) Cryo-EM density for gating residue interactions shown in **Fig. 3**.

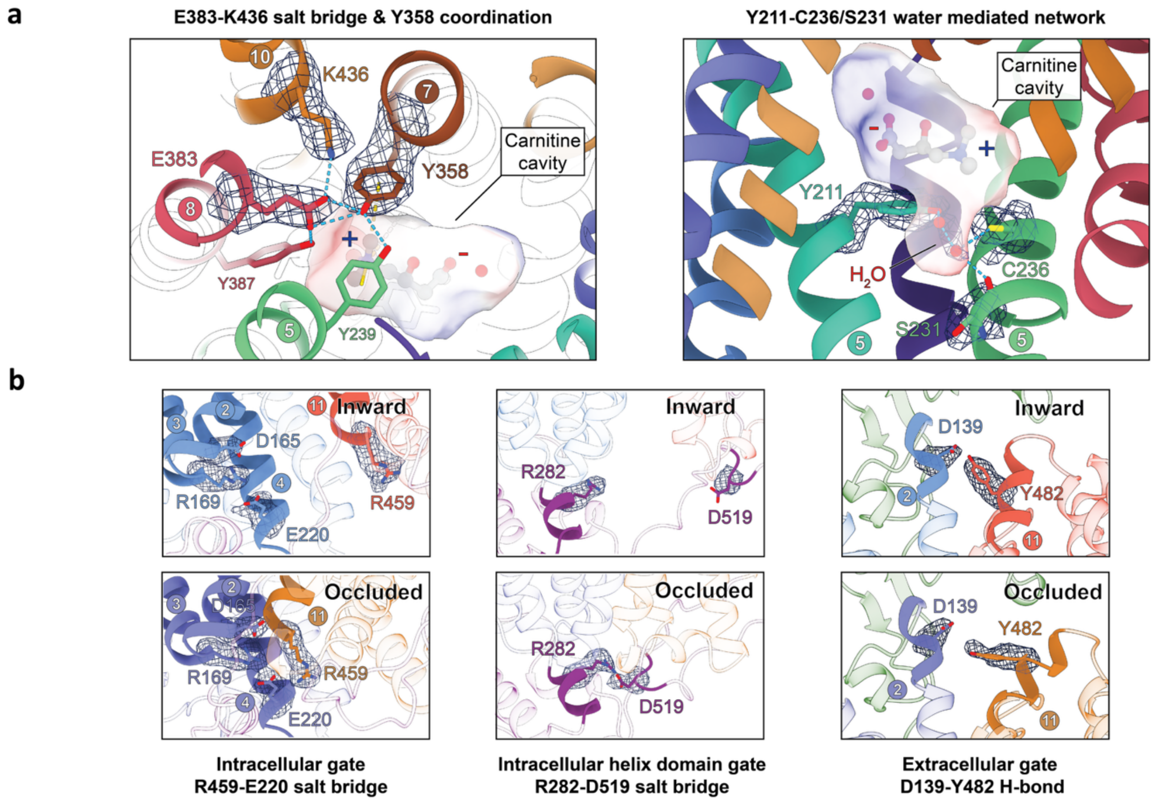

Supplementary Figure 6. (**a**) Comparison of the hOCTN2 Na^+ site and Alphafold3 modelling of the putative sites in hOCTN1 and mOCTN3. Predicted models are coloured by pLDDT score, as indicated by the key. Predictions were made using one sodium ion as an input. (**b**) Structural superposition of hOCTN2 (cryo-EM structure) and mOCTN3 (Alphafold3 prediction), and mOCTN3 coloured by pLDDT. (**c**) Comparison of binding site residues in hOCTN2 and mOCTN3. Residues conserved in both proteins are coloured purple, while those that vary are coloured cyan. The dual histidine/glutamine motif implicated in sodium dependence is highlighted.

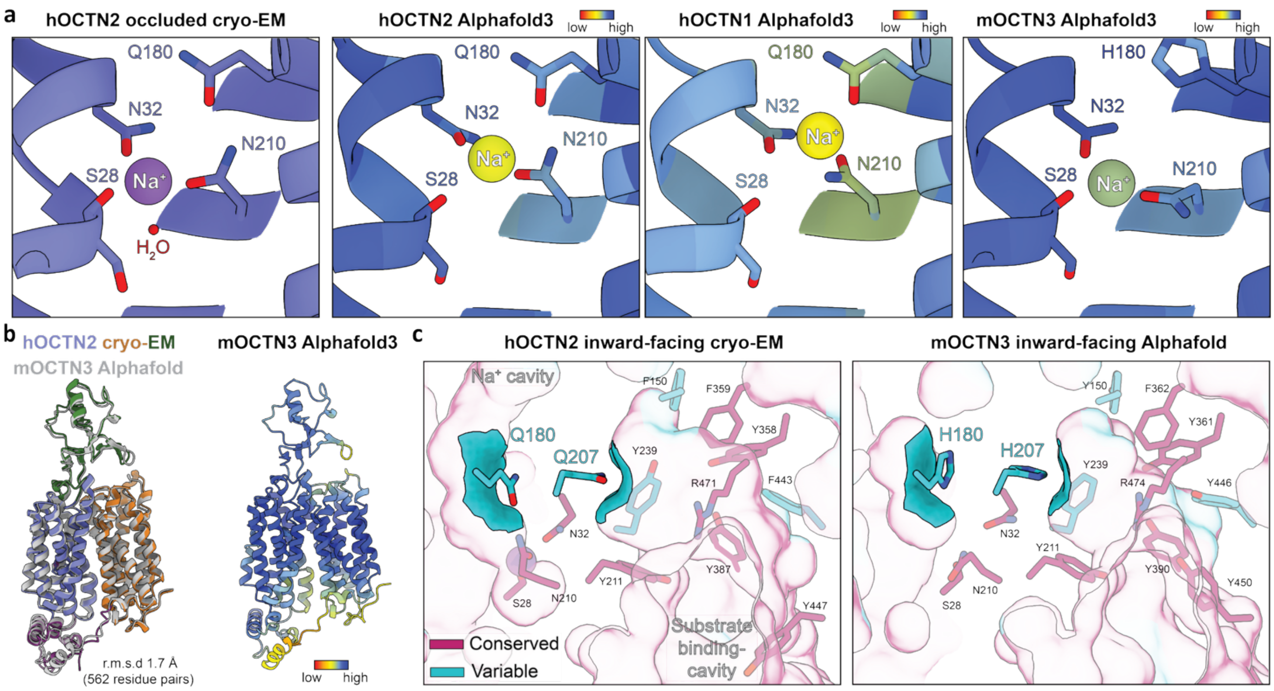

Supplementary Figure 7. Logarithmic plot showing Na^+ dependence of carnitine affinity. Logarithmic Na^+ (µM) concentrations were plotted against logarithmic L-carnitine K_M values (listed in Extended Table 2) and fit to a linear regression by method of least squares as shown by the graph (slope = -0.90 ± 0.1) Replicates were measured in at least five oocytes (n=5) across at least two batches of oocytes. Error bars represent ± SEM.

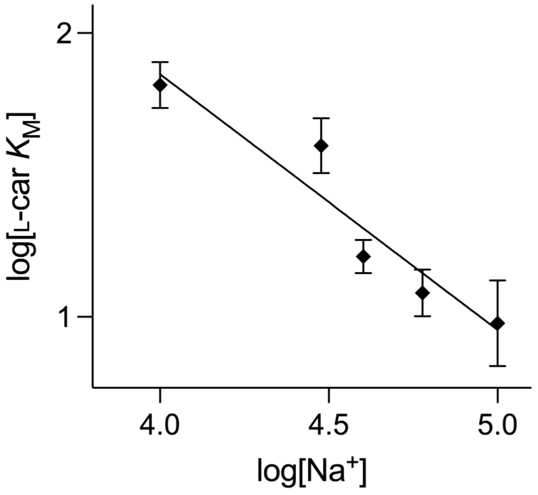

Supplementary Figure 8. Sequence alignment of human OCTs, OATs and murine OCTN3. Residues corresponding to TMs, ECD and ICH are depicted above the alignment. Carnitine-binding residues are indicated by ★ while Na^{+}-binding residues are indicated by *. Sequences were aligned using *Clustal Omega* and coloured in *Jalview* according to BLOSUM62 score.

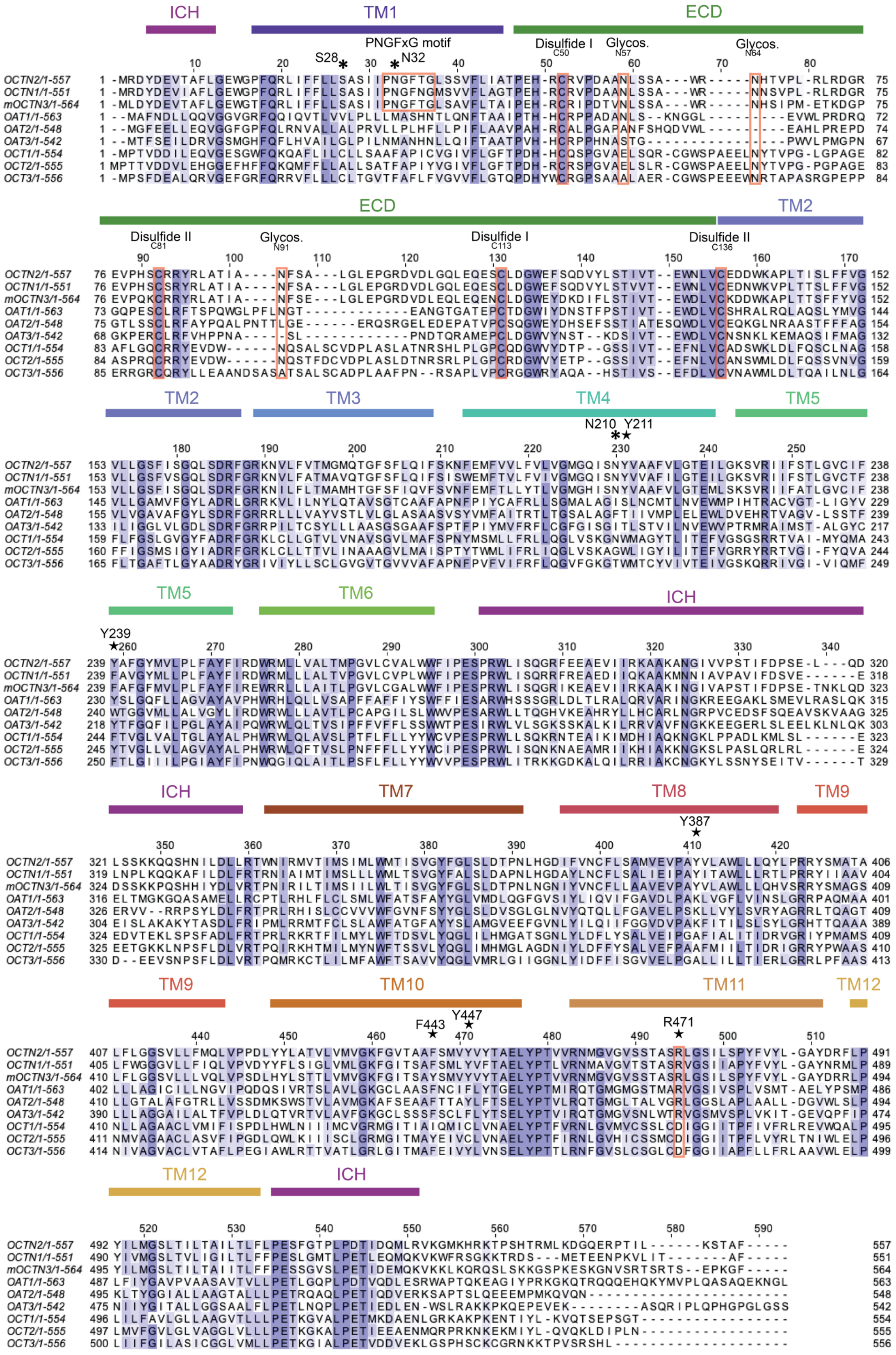

Supplementary Figure 9. Representative current traces for OCTN2 WT, mutant transporters and uninjected control oocytes. Oocytes were clamped at –60 mV and 100 μM L-carnitine was applied as indicated by the black bar.

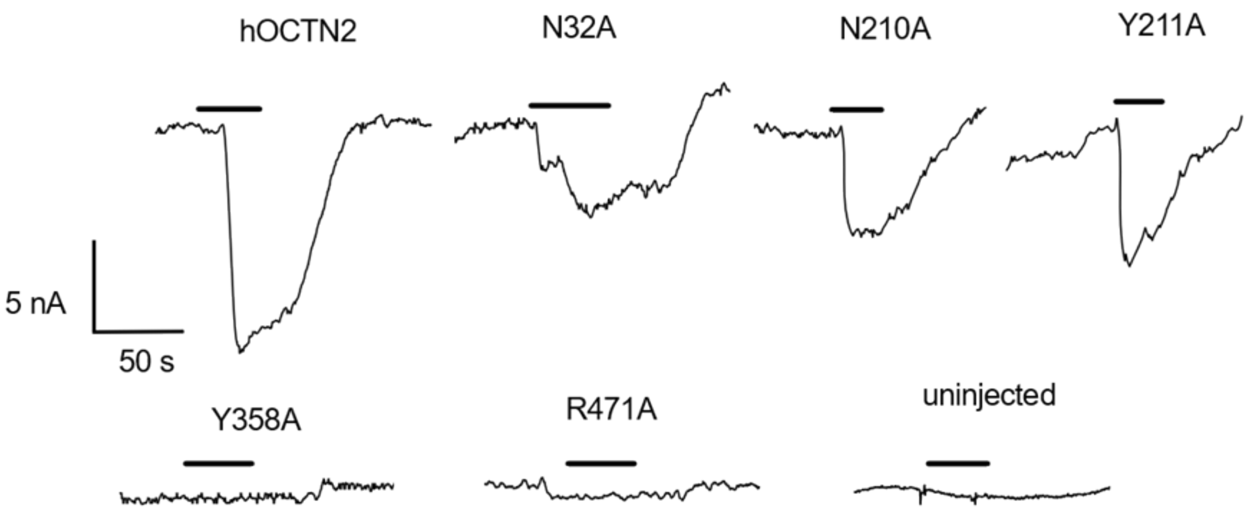

Supplementary Figure 10. (**a**) Ipratropium positioning within the occluded cavity upon structural overlay with the occluded conformation. (**b**) Comparison of the occluded carnitine-bound and the inward-facing ipratropium-bound conformations of OCTN2. Cut-through shows each conformation, with TM10 and TM11 shown as opaque helices. Call-outs show views of each binding site, with the ligands shown as ball-and-stick and the interacting residues shown as stick representation. Hydrogen bonds are shown in blue and cation-π interactions shown in yellow.

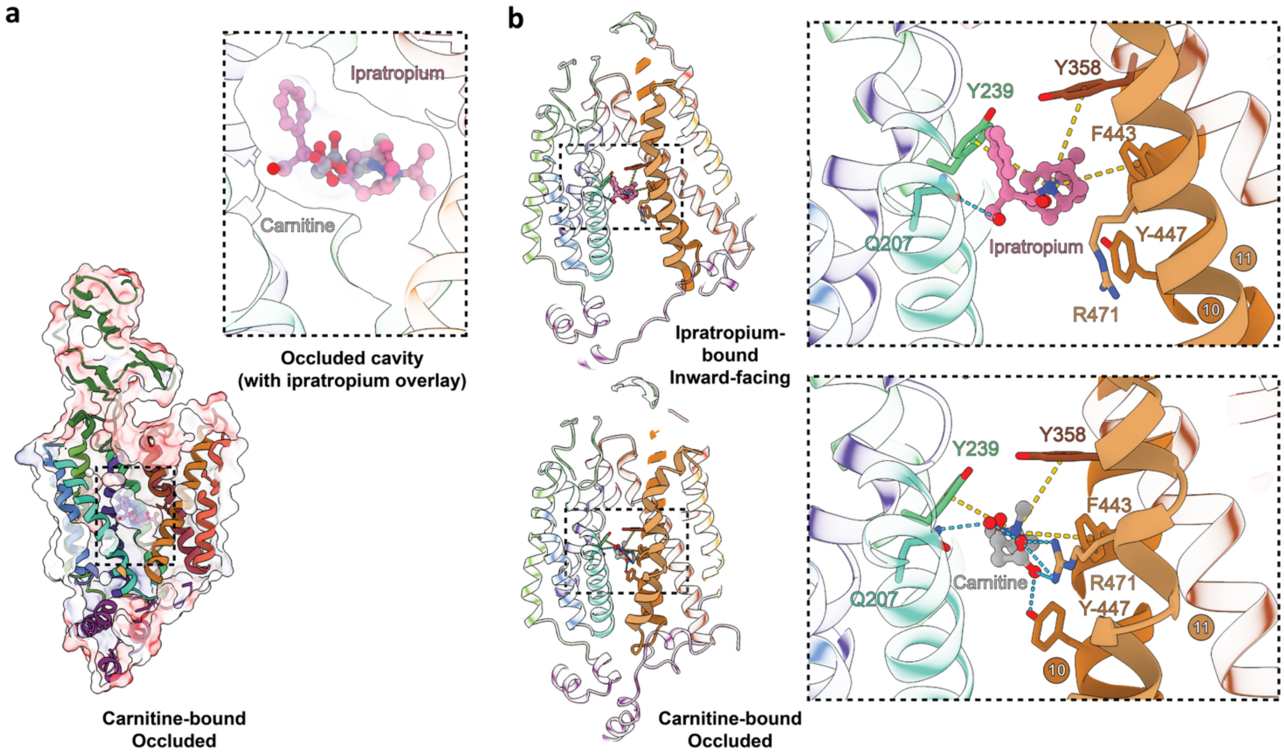

Supplementary Figure 11. Unprocessed SDS-PAGE gel image from hOCTN2 purification (Supp. Fig. 1)

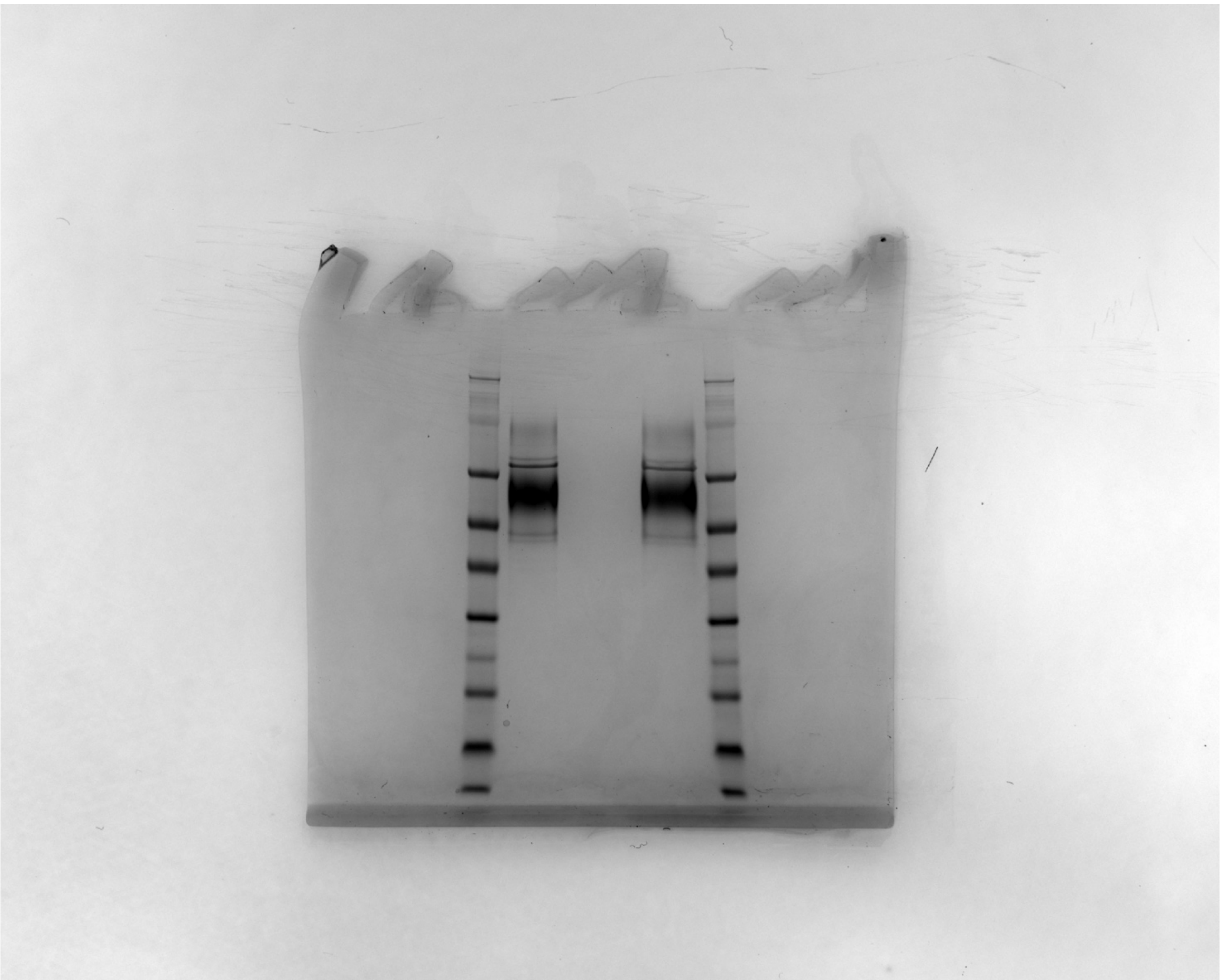

Supplementary Table 2. hOCTN2 L-carnitine affinities determined across a Na^+ concentration gradient. Replicates were measured in at least five oocytes (n=5) across at least two batches of oocytes. Error bars represent ± SEM.

$[Na^+]$ (mM)	L-carnitine K_M
10	69.7 ± 10.3
30	44.0 ± 8.8
40	16.9 ± 2.3
60	13.2 ± 3.1
100	12.0 ± 3.0

Supplementary Table 3. L-carnitine, Na^+ affinities and maximal currents measured for hOCTN2 mutant transporters. Replicates were measured in at least five oocytes (n=5) across at least two batches of oocytes. Error bars represent ± SEM. Na^+ affinity for N32A was unable to be determined. No reliable currents could be measured in Y358A or R471A.

	**L-carnitine K_M (µM)**	**Na^+ K_M (mM)**	**Hillslope of Na^+ curve**	**I_{max} at -60 mV (nA)**
WT	12.0 ± 3.0	8.62 ± 1.8	1.48 ±0.21	11.3 ± 2.1
N32A	210 ± 34	ND	ND	7.94 ± 1.2
N210A	131 ± 32	19.4 ± 7.0	0.91 ±0.11	10.8 ± 1.3
Y211A	260 ± 110	14.7 ± 5.3	1.16 ± 0.25	12.5 ± 2.5
Y358A	ND	ND	ND	ND
R471A	ND	ND	ND	ND
